# Shared governance increases marine protected area effectiveness

**DOI:** 10.1371/journal.pone.0315896

**Published:** 2025-01-08

**Authors:** Andrea Mast, David Gill, Gabby N. Ahmadia, Emily S. Darling, Dominic A. Andradi-Brown, Jonas Geldman, Graham Epstein, M. Aaron MacNeil

**Affiliations:** 1 Marine Affairs, Dalhousie University, Halifax, Nova Scotia, Canada; 2 Duke Marine Laboratory, Nicolas School of the Environment, Duke University, Beaufort, North Carolina, United States of America; 3 Ocean Conservation, World Wildlife Fund, Washington, DC, United States of America; 4 Marine Program, Wildlife Conservation Society, Bronx, NY, United States of America; 5 Center for Macroecology, Evolution and Climate, Globe Institute, University of Copenhagen, Copenhagen, Denmark; 6 School of Environment, Resources and Sustainability, University of Waterloo, Waterloo, Ontario, Canada; 7 Ocean Frontier Institute, Department of Biology, Dalhousie University, Halifax, Nova Scotia, Canada; Instituto Portugues do Mar e da Atmosfera, PORTUGAL

## Abstract

Marine protected areas (MPAs) are widely used to conserve and manage coastal resources. Protected areas are governed by a variety of institutional arrangements, yet little is known concerning the relative performance of different governance approaches. This research draws upon a unique dataset that combines details on the reported International Union for Conservation of Nature (IUCN) governance categories of 217 global MPAs and their ecological outcomes to compare the performance of alternative governance arrangements. We find that MPAs with shared governance arrangements, where management authority is shared among multiple government and non-government actors, are 98% more likely to have higher fish biomass than MPAs governed by state agencies (i.e., primarily government) alone (mean effect size and 95% C.I = 0.32 ± 0.31). We also find higher biomass in older MPAs, those in countries with higher gross domestic product (GDP), and those with a higher proportion of no-take area. With targets to protect 30% of our oceans driving new commitments to expand MPA coverage globally, our results suggest that multi-stakeholder participation and collaboration facilitated by shared and decentralized governance arrangements can play an important role in achieving conservation outcomes.

## Introduction

Marine protected areas (MPAs) are widely used to conserve and manage coastal resources [1, 2], as geographical spaces dedicated to the long-term conservation of nature and associated ecosystem services and cultural values [3]. Spatial management to reduce anthropogenic pressures on marine species and habitats can allow populations to recover from overexploitation [4], support important ecosystem functions and services [5, 6], strengthen ecosystem resilience [7–9], and in some cases, increase fish biomass in adjacent unprotected areas through spillover [10, 11]. Despite these benefits, protected area success is far from guaranteed, as outcomes depend on the design and operational rules of the MPA, as well as its staff and financial resources, which ultimately affect user compliance with rules [12–17]. Likewise, conservation outcomes are also heavily influenced by the governance processes and structures that are used in decision-making [18–21]. Some MPAs have weak or exclusionary governance structures that fail to incorporate the interests, values, and knowledge of local stakeholders in decision making [15, 22–25]. In other cases, MPAs miss opportunities to recognize and integrate existing customary practices, rights, or governance systems [26–28], highlighting the need for more equitable and effective solutions to improve management, and ultimately, provide social and ecological conservation benefits [12, 20, 29–32].

MPAs are managed through a diversity of governance approaches. MPA governance refers to the set of organizations, decision-making processes, and structures that shape the operational rules, rights, and responsibilities of actors within a spatially defined coastal or marine area [33–35]. IUCN and many experts in environmental governance theory and practice are particularly attentive to the locus of decision-making authority as a defining feature of different types of governance regimes [20, 33]. IUCN, for example, categorizes protected area (PA) governance into four major classes: (i) governance by state (national or sub-national), (ii) shared governance (collaborative or joint governance shared among multiple rightsholders and stakeholders), (iii) governance by private actors, and (iv) governance by Indigenous Peoples and/or local communities (Table 1) [30].

**Table 1:** IUCN governance categories. Broad and specific IUCN governance categories and the number of MPAs used in this study. No data were available for the *Private Governance* and *Governance by Indigenous Peoples and Local Communities* categories [36].

Broad Category	Description	Specific Category	Description	Number of MPAs in this study	Example
Governance by Government (State Governance)	Government body holds the authority over the governance of the MPA, determines its conservation objectives, and develops and enforces the management plan. There may not be a legal obligation to consult stakeholders.	National	Federal government, ministry or agency holds the authority over the governance of the MPA.	84	South Water Caye Marine Reserve (Belize)–governed by the Belize federal fisheries department [37].
		Sub-national	Local-level government, ministry or agency holds authority over the governance of the MPA.	71	Shoalwater Islands Marine Reserve–governed by the Government of Western Australia Department of Biodiversity, Conservation, and Attractions [38].
Shared Governance	Multiple rightsholders and stakeholders hold authority over the governance of the MPA and can influence decision-making.	Collaborative	Management authority shared among multiple government and non-government actors, with decision-making authority resting with one agency (can be government or non-government).	52	Fowl Cays National Park (Abaco, Bahamas)–governed by the Bahamas National Trust with a core inter-agency management team which requires public participation and stakeholder engagement. The Bahamas National Trust holds decision-making authority [39].
		Joint	Pluralist board or other multi-party governing body holds authority over the governance of the MPA.	10	Leone Pala Seasonal Management Area (American Samoa)–primarily governed by the American Samoa Coastal Management program within the Department of Commerce but other agencies contribute to governance (for example the Department of Marine and Wildlife Resources and the American Samoa Environmental Protection Agency). All new projects must include a public hearing to obtain stakeholder input [40].
Private Governance	Protected areas under individual, cooperative, NGO or corporate control and/or ownership.	N/A		0	Misool Marine Reserve (Indonesia)–privately funded marine reserve. Area is leased from the local community by the Misool Resort and Misool Foundation [41].
Governance by Indigenous peoples and local communities	Protected areas where the management authority and responsibility rest with Indigenous peoples and/or local communities through various forms of customary or legal, formal or informal, institutions and rules.	Indigenous peoples’ areas	Indigenous peoples’ areas and territories established and run by Indigenous Peoples	0	Gitdisdzu Lugyeks Marine Protected Area (Canada)–formally designated, implemented and governed by the Kitasoo/Xai’xais First Nation [42]
		Community conserved areas	Areas established and run by local communities	0	Villagers of Sawaieke District on Gau Island have established and govern a permanent community marine protected area [43]

Shared governance (e.g. co-management) PAs have gained prominence in recent years as a tool for resource management and biodiversity conservation [44–46]. Proponents suggest that shared PA governance addresses the limitations of state-based, private, or community governance by incorporating the interests, values, knowledge, and resources of diverse groups in the planning and implementation of PAs [30]. Meaningful participation of local stakeholders in conservation planning, for instance, may provide opportunities to reconcile biodiversity conservation with the interests, values, needs, and priorities of the actors most affected by protection [31, 47]. Stakeholder participation may allow conservation planners to draw upon local and traditional knowledge to define PA boundaries and conservation rules that are more reflective of local social and ecological conditions [20, 48–51]. It may also enhance the overall legitimacy of the PA, where the involvement of state and non-state actors contributes to higher levels of cooperation [52–54]. However, local stakeholders alone can sometimes struggle to respond effectively to complex conservation challenges and manage external threats, due to the scope of the resource conservation problems being beyond the ability of local institutions to deal with effectively [55–58]. As a result, shared governance arrangements that include diverse groups throughout the design and implementation process (e.g., planning, monitoring, adaptation) may foster collaboration, support community interests, instill responsibility across multiple groups, and increase management capacity by leveraging shared resources [56, 57, 59, 60]. Given the reported social and management benefits of stakeholder participation in protected area governance, we hypothesize that shared governance may subsequently lead to increased ecological performance in MPAs [12, 18, 61].

Despite the multiple expected benefits of shared governance, evidence of its effects on conservation outcomes mostly stems from research on terrestrial protected areas (e.g., [62, 63]). Limited evidence suggesting potential MPA benefits draws from case studies, expert elicitation, or reviews of case studies (e.g., [12, 18]). Additionally, the specific structure that state governance (e.g., sub-national vs national) or shared governance (multi-stakeholder body [joint] vs. single body that collaborates with others [collaborative]) takes may also result in different outcomes [30].

Here we explore the hypothesis that shared governance can contribute to greater ecological benefits than state-based governance, and further examine whether outcomes vary between more nuanced governance categories. Using a global dataset of ecological outcomes from 217 MPAs, and fish biomass differences as a proxy for MPA conservation impacts [15, 46, 64], we examine the performance of MPAs of different categories of governance arrangements, based on the categories defined by IUCN [30, 65]. Specifically, our objectives are to compare the differences in estimated ecological effects between MPAs with: (1) state-only versus shared governance (Model 1; Table 1), and (2) nuanced subcategories of governance arrangements, including national, sub-national, collaborative, and joint governance (Model 2; Table 1). We did not assess MPAs with private or Indigenous/community governance due to a lack of data corresponding to those governance categories.

This analysis extends existing research [15, 16, 18, 66] by explicitly examining differences in observed ecological outcomes from hundreds of MPAs with different forms of governance (n = 217 MPAs) across various social-ecological contexts. Whilst other global studies offer insights on how MPA management and design attributes affect MPA outcomes (e.g. age, enforcement levels, staff capacity [15, 16]), few, if any, specifically examine the influence of governance structures on outcomes across multiple contexts in hundreds of MPAs. Given goals for the expansion of MPAs to 30% of the ocean over the next decade [67], identifying effective forms of governance that may lead to ecologically and socially desirable outcomes is particularly timely and can inform current expansion efforts. Implementing MPAs that demonstrate ecological benefits while providing opportunities for social benefits through inclusive and shared governance are more likely to be widely adopted, helping scale more effective and equitable forms of marine conservation [68].

## Methods

### Ecological outcomes

We assessed ecological outcomes across different governance arrangements using a response variable of net fish biomass difference between MPA and similar non-MPA sites estimated in Gill et al. (2017), representing data from 217 MPAs across 37 different countries. In our analysis, we accounted for the nested nature of the data, with MPAs (n = 217) nested within countries (n = 37) and covariates present at both scales. Gill et al (2017) sourced these data from seven independent global and regional datasets, with survey data collected via underwater visual censuses of marine fish populations. Fish biomass is the total biomass of all recorded fish species (g/100m^2^), averaged across all transects at each site. Recorded species varied between datasets; therefore response ratios were only calculated among surveys collected using the same methodology. Biomass was calculated using individual body lengths and allometric length-weight data obtained from the data provider or FishBase [69]. To assess net differences in biomass, we define an MPA effect as the natural logarithm of the ratio of mean fish biomass (g/100m^2^) observed inside MPA sites relative to mean fish biomass in statistically matched sites outside MPA boundaries and/or before establishment: LnRR (1). Positive LnRR values indicate there was greater biomass inside the MPA compared to their statistically matched non-MPA sites and, thus, a positive effect of protection.


$$LnRR=\log\left(\frac{MPA\;biomass}{nonMPA\;biomass}\right)$$ (1)

Gill et al (2017) used statistical matching to account for selection biases in MPA placement, spatiotemporal dynamics of fish response to protection, and other social, ecological, and physical factors that can affect fish populations that vary between sites [70–72]. Observed factors accounted for in the matching model include habitat type, distance from shore and population centers (“markets”), neighboring human population density, ocean conditions (e.g., chlorophyll concentration, wave energy, sea surface temperature), survey depth, survey location and year, country, and ecoregion [15]. By reducing the confounding effects of these factors, we are better able to isolate differences attributable to the MPA [70]. The final dataset of matched LnRR values was also supplemented by LnRR values from a global meta-analysis (n = 29) of MPA outcomes [73, 74], bringing the total to 217 MPAs. See Gill et al. (2017) supplementary material for more information on data sources and matching methods.

### MPA governance categories

In this study we used two separate models to examine the ecological effects of different forms of governance. In the first model (Model 1), we examined differences in ecological outcomes between MPAs with state and shared governance arrangements (Table 1). These two categories were broken down into subcategories in the second model, with state governance split into national and sub-national, and shared governance split into collaborative and joint governance (Model 2). While more nuanced governance structures and processes exist, these categories nonetheless provide an opportunity to examine the ecological performance of specific clusters of MPA governance types.

We sourced MPA governance types from the November 2017 version of the World Database on Protected Areas (WDPA). Where required, we supplemented these data using MPAtlas, the Caribbean Marine Protected Area Management (CaMPAM) MPA database, and MPA-specific management documents [75, 76]. We validated information sourced from the WDPA using management plan documents, government websites, and scientific papers. Care was taken to identify governance conditions at the time of the fish survey sample. In some cases no secondary sources were found, and the governance categorization listed in the WDPA was used. The validation process led to 20 MPAs changing subcategories (e.g. joint to collaborative) and 13 changing broader categories (e.g. collaborative to national). We re-ran the models using only validated governance categories, and the outputs remained unchanged, indicating that the results were not sensitive to re-categorizations.

### Model covariates

Covariates used in this study are summarized in Table 2. We standardized all covariates by subtracting the mean and dividing by two times the standard deviation to ensure effect magnitudes were comparable across the different covariates [77]. We then used the mean-centered covariates to estimate the increase in biomass ratio for each unit change in covariate using a Bayesian hierarchical model, described in the ‘statistical analysis’ section. We checked correlation between covariates using a Pearson correlation test before running the analysis. Gross domestic product (GDP) and World Bank governance indicators (WGI) were positively correlated (0.85) while human development index (HDI) and the fish catches per capita were negatively correlated (-0.75; S1 Fig). While the models account for covariate correlation, we ran a separate model without the correlated covariates that resulted in minimal changes to the results (S1 Table).

**Table 2:** Model covariates. Summary information on the covariates used in the model.

Covariate Name	Scale	Description	Range	Year
MPA Governance	MPA	MPA governance type.	Shared (collaborative or joint); state (national or sub-national).	Varied based on the year of MPA establishment and date and location of survey data collection [15].
MPA age (years)		Number of years the MPA has been established.	1–95 years	
Distance from shore (km)		Average distance of MPA survey sites from the nearest shoreline.	0–95,000 km	
MPA size (km^2^)		Size of the MPA in square kilometers.	0.01–35,000 km^2^	
No take		Portion of fish surveys conducted in no-take zone (indicating no-take zone or MPA).	0–1	
National governance	Country	Captures six key dimensions of governance determined by the World Bank including voice & accountability, political stability and lack of violence, government effectiveness, regulatory quality, rule of law, and control and corruption. The six indicators were averaged into a composite indicator.	-0.9–2.3	2005
Gross domestic product		Total market value of the goods and services produced by a country’s economy per year.	41 million– 4.9 trillion	2005, 2006
Human development index		Summary measure of average achievement in key dimensions of human development including long and healthy life, being knowledgeable, and having a decent standard of living.	0.418–0.939	2005, 2008
Fish catch per capita per EEZ (tons)		Total weight of fish catches (in tons) at the time of landing, per capita.	125 tons– 6.4 million tons	2005
Population largest nearby city		Total population count of the largest city located within 100 km of the MPA.	138 individuals– 4.9 million individuals	2005

#### MPA level covariates

We obtained MPA level covariates from the Gill et al. (2017) dataset to account for their effects on outcomes. This includes the average distance from shore, size, and age of each MPA, as well as the proportion of the survey sites sampled from no-take zones within the MPA (range: 0–1). Here MPA governance age, regulations (e.g. no take), and distance from shore for each survey site was based on the year of establishment and the date and location of survey data collection (respectively); see Gill et al. (2017) for more information.

#### Country level covariates

We averaged six indicators of broad dimensions of national governance from the World Bank into a composite indicator for each country and used as a country-level covariate: (1) voice and accountability, (2) rule of law, (3) control and corruption, (4) political stability, (5) government effectiveness, and (6) regulatory quality [78]. Descriptions of each governance indicator, and more information on how the composite indicator was determined, can be found in the supplementary information (S1 File). Other country-level covariates included were GDP and HDI. We sourced GDP and HDI data from the World Bank [79]. Where possible we used data that matched the median year of ecological data collection, which was 2005. Where data from 2005 was not available, we used the closest year with data (see S1 File for more information).

We also examined fish landings per capita per Exclusive Economic Zone (EEZ) area and the population of the largest city within 100km. We obtained the fish landings from the Food and Agriculture Organization that correspond to the total catches (in tons) [80]. We obtained the population of each country and city within 100km through the World Population Review [81], and EEZ area through ESRI’s database [82].

### Statistical analysis

To quantify the multi-scale factors affecting MPA impacts we adopted a Bayesian hierarchical modelling approach (2). We developed a null model and used it as a baseline to assess the performance of the covariate model relative to a model that only accounts for the inherent hierarchical structure of the data. Using a multilevel model allows for the recognition of both local and national level factors and accounts for error at both levels. We entered covariates into the models at their appropriate scale, with lower level (MPA) covariates nested within higher level (country) model intercepts to address potential pseudo-replication from the nested structure of multiple MPAs within countries. The two full models assumed that country (*μ*_*i*_) and MPA (*η*_*ij*_) level outcomes were normally distributed, given a uniform prior for the standard deviation (*σ*_*β*_, *σ*_*γ*_):

$$Y_{ij}∼N(\eta_{ij},\sigma_{\beta })$$

$$\eta_{ij}=\beta_{0i}+\beta_{1}*Z_{1ij}\ldots +\beta_{n}*Z_{nij}$$

$$\beta_{0i}∼N(\mu_{i},\sigma_{\gamma })$$

$$\mu_{i}=\gamma_{0}+\gamma_{1}*X_{1i}\ldots +\gamma_{n}*X_{ni}$$

$$\gamma_{0\ldots n},\beta_{0\ldots n}∼N(0,100)$$

$$\sigma_{\gamma ,}\sigma_{\beta }∼U(0,100)$$ (2)

Where *β* are the covariate coefficients of *Z* MPA level covariates, *γ* are the country level covariate coefficients of *X* country level covariates, and *Y* is the response variable, or fish biomass difference, for *j* MPA in *i* country. Estimation was carried out using the PyMC3 package [83] for the Python programming language. We conducted posterior predictive checks for goodness of fit by examining posterior predictive distributions for the observations, checking Geweke scores from multiple chains for each parameter, and from observed fits of the model and data [84]. We found no evidence of poor model fit for either model, with posterior predictive distributions consistent with the observed data (S2 File). Geweke Z scores from observed fits of the model and data was 0.47 for both models, indicating a good fit (S3 File).

With the appropriate model structure defined, we developed the general (Model 1: state and shared governance) and sub-group (Model 2: collaborative, joint, sub-national, and national governance) models to compare the relative effects of different forms of governance. For the categorical governance variables, the model output is the difference from the baseline category, state governance in Model 1 and national governance in Model 2.

## Results

**Fig 1:**
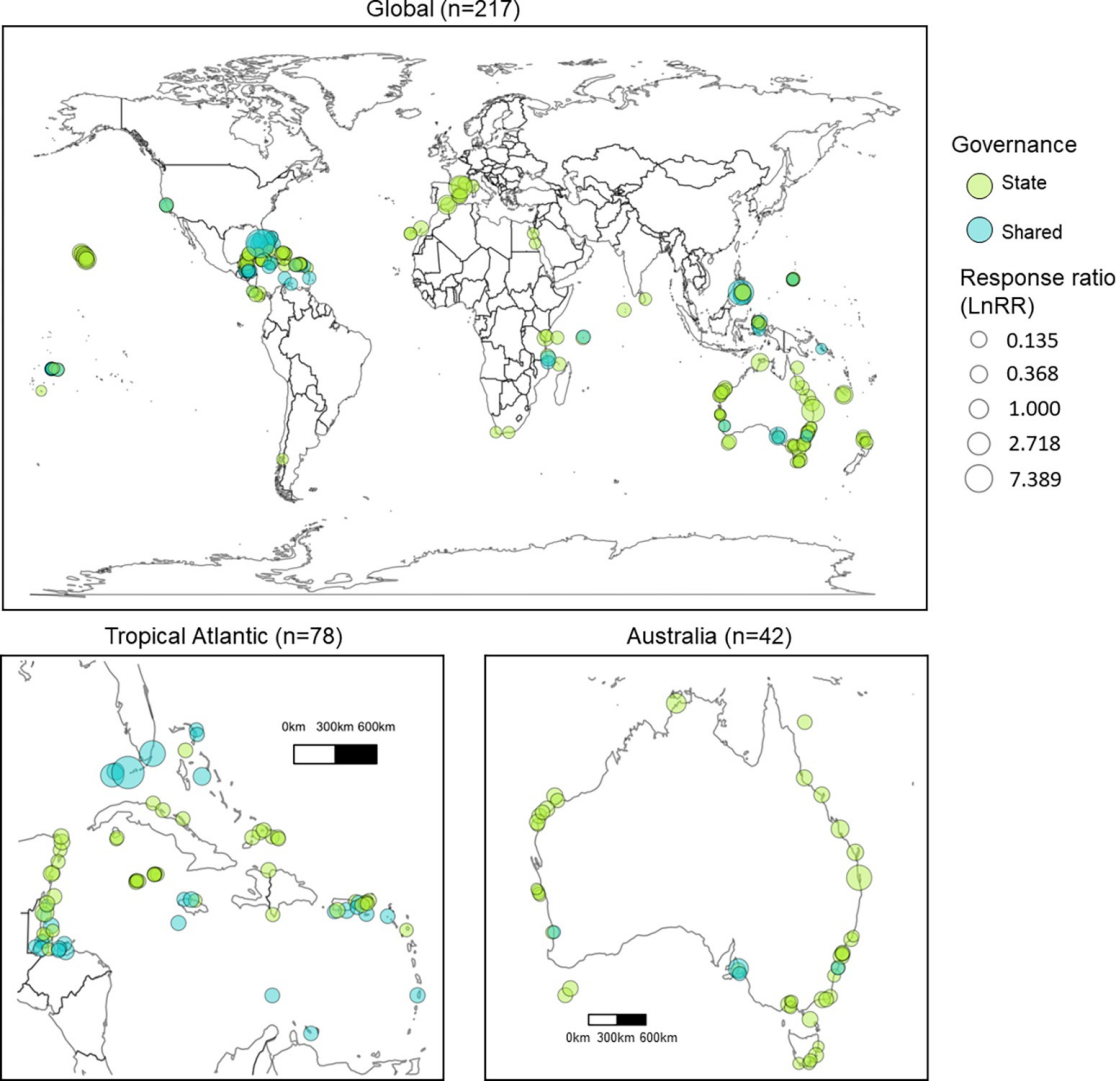
Global distribution of MPA governance categories. World map depicting the 217 MPAs used in this study. The color of the circle represents the governance of the MPA (state or shared from Model 1) and the size of the circle represents the corresponding response variable, or fish biomass difference, for each MPA. The response variable was transformed to ratios from log ratios prior to mapping to more accurately display the differences of each MPA.

The net difference in fish biomass associated with MPAs, LnRR, ranged from -3.76 to 3.70 (mean: 0.47; standard deviation: 0.96). Of the 217 MPAs in this study, 155 were classified as state governance (84 national and 71 sub-national) and 62 as shared governance (52 collaborative and 10 joint). MPAs were distributed worldwide, with both shared and state governance present in each country represented in the data (Fig 1). On average, the MPAs in our sample had been established for a mean of 17 years (range: 1 to 95 years) with a mean area of 610 km^2^ (range: 0.01 to 35,000 km^2^) (Table 2). MPAs in the sample included those that prohibit all fishing activity (i.e., "no-take”) and those where fishing was allowed in some or all areas within the MPA. In this study, 127 MPAs (59%) had fish survey data from no-take zones or no-take. Of these, 91 exhibited state governance and 36 exhibited shared governance.

**Fig 2:**
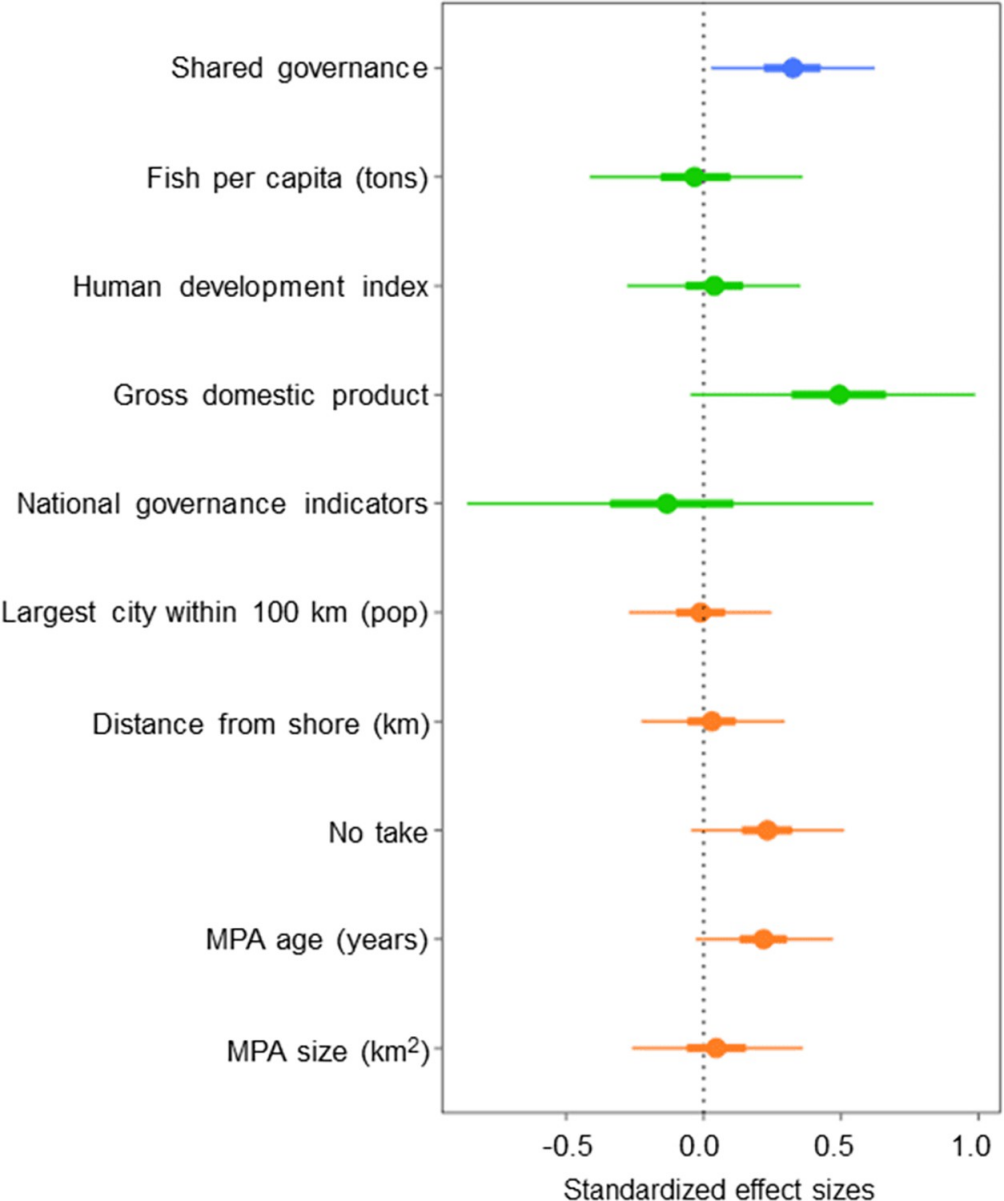
Effects of Model 1 covariates on fish biomass. MPA governance (blue), country characteristics (green), and MPA characteristics (orange) on fish biomass difference. Response variables are log fish biomass differences and represent Bayesian posterior mean effect sizes (dot) with 95% Bayesian credible intervals (C.I.s, thin lines) and 50% C.I.s (thick lines). The baseline governance category in this model was state governance. The national governance indicators represent the World Bank governance indicators. No-take refers to the proportion of fish surveys conducted in no-take zone. See Table 2 for variable descriptions.

MPAs with shared governance had greater fish biomass than those with state governance (Bayesian posterior mean effect size and 95% confidence interval (C.I.) = 0.32 ± 0.31) (Fig 2 and Table 3). While both, on average, provided biomass benefits, MPAs with shared governance provided biomass benefits that were on average 32% greater than those provided by state governance (Table 3) and there was a 98% chance that an MPA with shared governance would have greater fish biomass than state governance (P(shared>state) = 0.98; S2 Fig).

**Table 3:** Model covariate effect sizes. Bayesian posterior mean effect size and 95% credible intervals (C.I.) for each covariate used in both models.

Covariate	Mean effect size and 95% C.I.	
	Model 1	Model 2
MPA size (km^2^)	0.056 ± 0.30	0.061 ± 0.31
MPA age (years)	0.21 ± 0.24	0.24 ± 0.24
No take	0.22 ± 0.28	0.23 ± 0.28
Distance from shore (km)	0.03 ± 0.26	0.05 ± 0.25
Largest city within 100 km (pop)	-0.011 ± 0.25	-0.04 ± 0.26
National governance indicators	-0.11 ± 0.80	-0.13 ± 0.77
Gross domestic product	0.48 ± 0.63	0.39 ± 0.63
Human development index	0.026 ± 0.33	0.046 ± 0.32
Fish per capita (tons)	-0.032 ± 0.60	-0.11 ± 0.61
Shared governance	0.32 ± 0.31	N/A
Collaborative governance	N/A	0.49 ± 0.35
Sub-national governance		0.26 ± 0.37
Joint governance		0.21 ± 0.65

**Fig 3:**
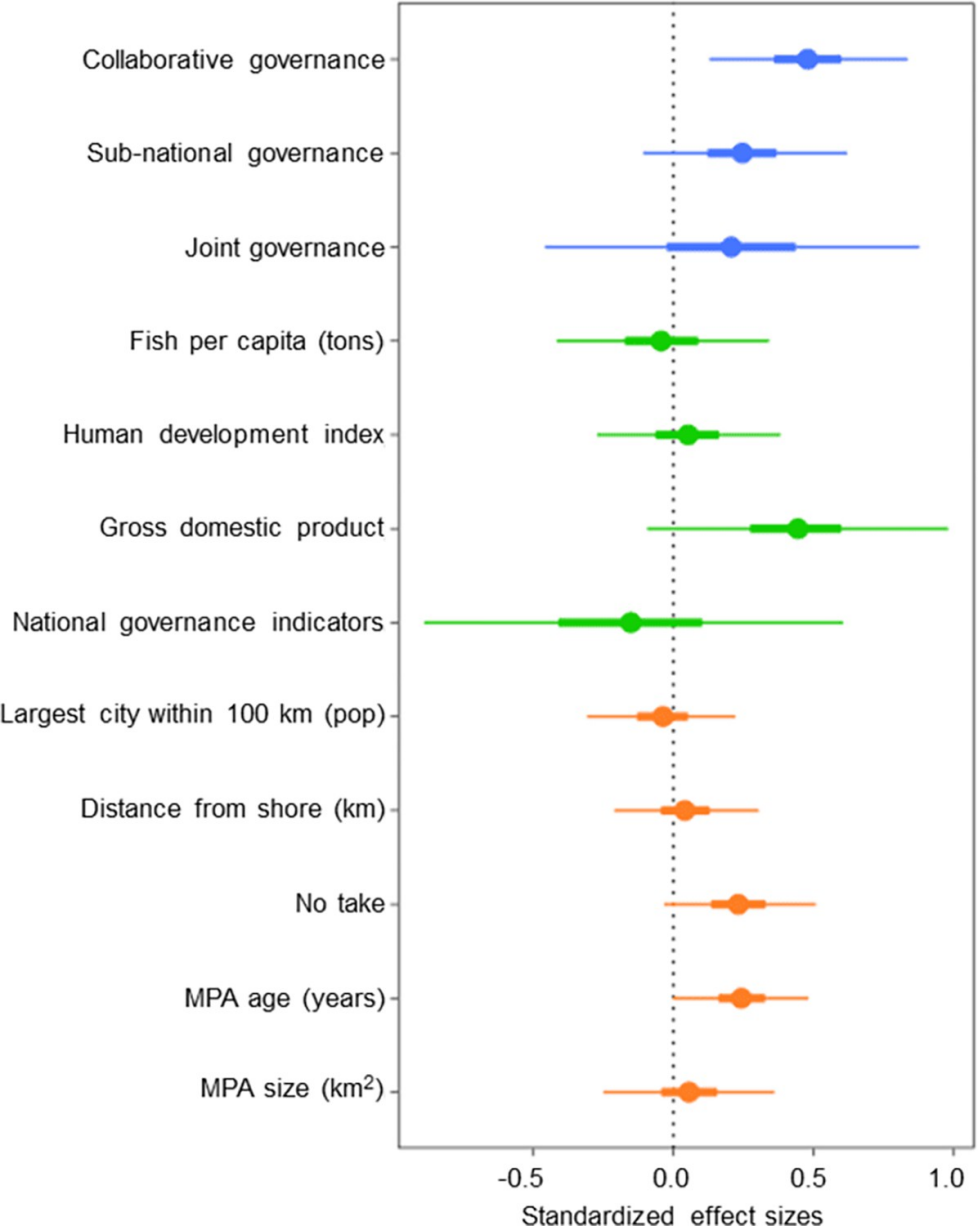
Effects of Model 2 covariates on fish biomass. MPA governance (blue), country characteristics (green), and MPA characteristics (orange) on fish biomass difference. Response variables are log fish biomass differences. Represents Bayesian posterior mean effect sizes (dot) with 95% Bayesian credible intervals (C.I.s, thin lines) and 50% C.I.s (thick lines). The baseline governance category in this model was national. The national governance indicators represent the World Bank governance indicators. No-take refers to the proportion of fish surveys conducted in no-take zone. See Table 2 for variable descriptions.

When looking at governance structures at a higher resolution, subnational, collaborative, and joint governance types all had a positive effect on fish biomass difference compared to nationally managed MPAs with collaborative governance having the greatest positive effect (Bayesian posterior mean effect size and 95% C.I. = 0.49 ± 0.35), followed by sub-national (0.26 ± 0.37) and joint (0.21 ± 0.65) (Fig 3; Table 3). With national governance as the baseline category in Model 2, collaborative, sub-national, and joint governance supported biomass levels that were on average 49%, 26%, and 21% higher than those supported by national governance respectively (Table 3), and were 99%, 91%, and 73% more likely to do so (S3 Fig).

At the MPA level, MPA age had a positive effect on fish biomass (Bayesian posterior mean effect size and 95% C.I. = 0.21 ± 0.24 in Model 1 and 0.24 ± 0.24 in Model 2) as well as the proportion of MPA samples taken from a no-take zone (0.22 ± 0.28 for Model 1 and 0.23 ± 0.28 for Model 2). Each unit change in the age of the MPA provided on average a 21% and 24% change in the biomass ratio in Models 1 and 2, respectively. Similarly, each unit change in the proportion of no-take zone sites provided on average a 22% and 23% change in the biomass ratio in Models 1 and 2, respectively, suggesting a positive effect from no-take regulations (Table 3). At the country level, the gross domestic product (GDP) had a positive effect on fish biomass (0.48 ± 0.63 in Model 1 and 0.39 ± 0.63 in Model 2). The biomass ratio changed by an average of 48% and 39% per unit change in GDP in Models 1 and 2, respectively (Table 3). No other MPA and country level covariates had a significant effect on fish biomass.

## Discussion

Our results show greater biomass increases in MPAs with shared versus national-level state governance arrangements. These findings support a broad and growing set of theoretical and empirical literature linking the inclusion of participatory and decentralized governance to sustainable outcomes [20, 31, 47, 85, 86], including several recent reviews and multi-site assessments of conservation and fisheries management interventions [18, 61, 87, 88]. For example, inclusive governance structures such as co-managed MPAs and fisheries have been linked to improved mangrove forest conditions [89], improved benthic habitat conservation [90], and other related outcomes such as increased compliance with regulations [91, 92], and fisher perceptions of improved catch [93, 94]. With our study representing a diversity of MPAs with differing design attributes and management goals, our results add to this body of literature showing that participatory and decentralized governance, which can take many forms, are likely to play a key role in determining conservation outcomes across multiple contexts. In some cases, shared governance can include multiple actors in management with no explicit commitment to non-government actors, or actors at the local community level, as is the case with the joint governance category explored in Model 2 of this study. A potential lack of commitment to non-government actors may explain the underperformance of the joint governance category compared to the collaborative governance category, both of which fall under the broader shared governance category.

While the results are clearly supportive of the general hypothesis that inclusive governance structures support better ecological outcomes for MPAs, they also show that governance led by sub-national units (i.e., local, provincial governments) tend to outperform their national-level counterparts when it comes to fisheries biomass. In theory, much like with shared governance, shifts towards lower levels of government can help to develop rules that are better adapted to local conditions [20] and enhance the overall legitimacy of rules and governance systems by reducing the distance between decision-makers and the communities affected by those decisions [95, 96]. Although the empirical evidence regarding the relationship between sub-national governance and environmental outcomes is decidedly mixed [97, 98], this study provides at least some evidence to suggest that sub-national governance may be better situated to manage MPAs to deliver ecological outcomes than their national-level counterparts.

While other MPA attributes and national contexts help shape MPA outcomes, the majority of these factors were not as important as governance in explaining outcomes in our data. Our models showed that older MPAs had higher fish biomass, a finding that is well established [4, 13, 16]. Older MPAs give slow-growing species more opportunity to recover [4, 99, 100] and give managers more time to implement and adapt management activities [13, 101, 102]. Also, our results suggest that no-take zones or no-take MPAs had higher fish biomass, however, the effect was not as strong as expected from previous work [73, 103–105]. In Edgar et al (2014), MPAs with no-take regulations had greater differences in multiple outcome metrics (e.g. total biomass, exploitable fish biomass, etc.) compared to sites that allowed fishing. Nonetheless, the effect of shared governance in this study was over 1.5 and 1.4 times greater than the effects of MPA age and proportion of no-take sites in explaining variation in outcomes. Further research using causal inference approaches on the interactive effects of different types of governance, MPA age, and fishing regulations is warranted [106].

At the country level, GDP had a positive effect on fish biomass. These countries may have greater resources for environmental management that allows for greater marine conservation funding, leading to greater capacity for MPA governance and management [107] as well as greater education [108]. However, large investments do not always guarantee success of a protected area as other factors may influence conservation outcomes such as management efficiency [109]. In addition, conservation funding is often unequally distributed within and between countries [110]. Our models showed little predictive power between the other country-level covariates and fish biomass. This is likely because variation in MPA outcomes between countries is much lower than differences between individual MPAs. Other studies have found very weak relationships between country level factors and local conservation activities [15, 111], highlighting the role that local context plays in determining conservation outcomes [36].

### Implications for management

This study addresses an important gap in the MPA literature concerning the impacts of alternative governance structures on biodiversity conservation and highlights opportunities for conservation to deliver ecological benefits through shared governance. The results further suggest that in cases where there are barriers to developing shared governance arrangements, that MPAs governed by decentralized governments may deliver more ecological benefits than their national-level counterparts.

With increased calls for more effective and equitable conservation, collaborative and localized governance arrangements provide a more appropriate model for the next generation of biodiversity conservation to support social and ecological conservation goals [32, 67]. Sub-national governance can facilitate MPA design and management more suitable to the local social-ecological context than centralized systems as well as more direct engagement with local actors [31]. Collaborative governance arrangements that facilitate the inclusion of diverse voices, perspectives, aspirations, and types of knowledge in the decision-making process can support key elements of equitable conservation, namely: recognition of user rights, participatory decision-making, and more equitable distribution of benefits [20, 49, 112–114]. Shared governance can facilitate the articulation of user rights, values, and interests, and shed light on local socioeconomic conditions (e.g. resource-dependency and customary practices) which are essential to mitigate the potential negative effects on local resource users or to identify necessary compensatory mechanisms [14, 30, 115–117]. For example, on the island of Tonga co-management created a greater sense of ownership and a greater sense of involvement in the management of resources for ‘O’ua fishers. When interviewed, they reported improved socio-economic conditions and increased income following the implementation of co-management, which gave ‘O’ua fishers exclusive fishing rights to the reefs adjacent to the island [118].

While some nationally managed MPAs allow stakeholder input in design and planning (e.g., the Northern Shelf Bioregion MPA network in Canada [119]), shared governance arrangements are more conducive to pluralistic decision-making throughout the management cycle (e.g., planning, monitoring, enforcement, and adaptation) [112, 120, 121], encouraging collaborative learning and adaptation. Additionally, the inclusion of multiple actors and institutions can create more resilient governance systems, where periods of instability or failure in one institution can be buffered by another [30, 122]. With the potential for greater inclusivity, local ownership, cooperation, management capacity, and resilience [44, 51, 123–125], as well as greater ecological outcomes, collaborative and decentralized governance approaches appear to provide the enabling conditions for achieving more effective and equitable conservation. This is the case in American Samoa, where a Community-based Fisheries Management Program, which deputizes local villagers as enforcement agents, has been implemented in some areas. The program is based on traditional Samoan systems of marine tenure and provides government support through formalized management plans and legislative backing for the areas through regulations [40]. By working through traditional Samoan village systems to establish marine regulations village residents are more likely to comply with rules and regulations, and are motivated to monitor the waters adjacent to their land [40].

We recognize that devolved governance arrangements such as co-management are not a panacea, and that shared governance can also lead to negative outcomes if not implemented correctly [59]. Cases exist where *de jure* shared arrangements differ from what occurs on the ground, and many stakeholders remain detached from the management process [126, 127]. In other cases, shared governance provided additional power to outside organizations or elites within a community, exacerbating existing inequalities [22, 128, 129]. As with any management system, positive conservation and social outcomes require well-designed and contextually appropriate interventions where governance principles such as (*interalia)* transparency, accountability, fairness, legitimacy, and inclusion are adequately applied [21, 31, 36, 130].

### Future research

Our results support a large and growing body of research highlighting the role of governance as a leading driver of conservation outcomes, and opportunities to support biodiversity conservation though the adoption of institutional arrangements that foster collaboration and power sharing among multiple stakeholders [18, 20, 44, 112, 131–134]. However, it is important to note that there are several limitations associated with this analysis. First, although the IUCN governance categories provide a consistent framework for distinguishing major classes of MPA governance, there remains considerable diversity within each category. For example, shared governance which is often envisioned as collaborations between governments and local stakeholders may also include arrangements in which governments collaborate instead with multiple non-local actors (e.g., foreign NGOs) or simply consult local stakeholders with no delegation of decision-making authority [135]. On the other hand, state governance can include MPAs that allow stakeholder consultation and input in management decisions, or delegate certain management activities to local actors [65]. Further research on the outcomes from these more nuanced forms of governance is warranted [136, 137]. Changes in governance structure just before field sampling could also affect the results, as it is not clear how long particular governance structures need to be in place before it affects ecological conditions. Furthermore, because of data limitations, we were unable to assess the effect of private or Indigenous/community governance on ecological outcomes, and we suggest that future studies that assess the relative impacts of MPA governance include those managed solely by local non-state actors.

To fully assess the role that participatory governance may play in ecological outcomes, future studies should adopt counterfactual approaches, comparing outcomes of participatory MPAs to outcomes had that same MPA not been participatory [72, 138]. In this study, our ability to isolate MPA effects was limited by the availability of baseline data, which highlights the need for improving MPA monitoring and evaluation to support causal inference research. Additionally, important questions remain concerning the mechanisms by which shared and decentralized governance emerge and shape ecological outcomes [139], and the factors driving synergies and/or tradeoffs between social and ecological outcomes under various governance arrangements. Research on the interactions between governance and other factors such as MPA design attributes (e.g., size, configuration, and fishing restrictions) and social-environmental context will help to shed light on the enabling conditions for outcomes and advance theory on protected area governance.

## Conclusion

Our research suggests that shared and decentralized governance can play a measurable role in achieving improved ecological outcomes. In addition to greater conservation outcomes, devolved and inclusive governance can help to promote social justice and cohesion, the rights of marginalized groups, and increase management capacity and resilience, providing social benefits and contributing to multiple development and conservation goals [30, 67, 140, 141]. Given the potential social and ecological benefits, managers can make a concerted effort to include diverse stakeholders and form partnerships with local groups throughout the management process (e.g., design, implementation, management, adaptation) to more effectively and equitably protect their coastal environments. Nonetheless, shared and decentralized governance are not panaceas, and requires appropriate investment of time and resources to ensure participatory and representative governance within the MPA design stage and beyond. This includes a careful assessment of the preexisting social context, the diversity of rightsholders and stakeholders and capacity to participate in management, and pre-existing resource use patterns and management [24, 30, 132]. With the expected rapid increase in MPAs to meet global conservation targets [67], our results suggest that governance arrangements that include multiple stakeholders in the management process are likely to provide greater ecological benefits than potentially less inclusive, centralized approaches and foster more effective and equitable conservation.

## Supporting information

S1 File Additional information on country level covariates. (DOCX)

S2 File Posterior predictive distributions. (DOCX)

S3 File Posterior predictive distribution of the mean and Geweke Z scores. (DOCX)

S1 Fig Correlation matrix–raw data. (DOCX)

S2 Fig Shared governance posterior density. (DOCX)

S3 Fig Collaborative, sub-national and joint governance posterior densities. (DOCX)

S1 Table Covariate mean effect sizes. (DOCX)
