# Supplementary material for: Shared governance increases marine protected area effectiveness
Source: PLoS One. 2025 Jan 8;20(1):e0315896. doi: 10.1371/journal.pone.0315896 (PMC11709245; doi:10.1371/journal.pone.0315896)
Supplement: S1 File — (DOCX) [file pone.0315896.s001.docx]

**S1 File. Additional information on country level covariates**

**Gross Domestic Product and Human Development Index**

Gross Domestic Product (GDP) values from 2005 were gathered from the World Bank. The following countries did not have GDP information for 2005, so data from 2006 was used: Cayman Islands, New Caledonia, Curaçao, Saint Lucia, and Turks and Caicos. HDI values from 2005 were gathered from the United Nations Development Program Human Development Reports. The following countries did not have 2005 data, so data from 2008 was used: Cayman Islands, Curaçao, New Caledonia, Puerto Rico, Saba, US Virgin Islands, Puerto Rico, Guam, and Turks and Caicos Islands.

**World Governance Indicators**

The World Governance Indicators (WGI) are a dataset by the World Bank summarizing the views on the quality of governance. The World Bank sources these data from a large number of enterprises and citizen and expert survey respondents in industrial and developing nations to determine six aggregate governance indicators ranging from -2.5 to 2.5. We used data from 2005 as it was the median year for ecological data collection, and we averaged all six governance indicators into one composite indicator for each country used in the study. The six World Bank Indicators are summarized below.

| **Metric** | **Description** |
| --- | --- |
| **Voice and accountability** | Reflects perceptions of the “extent to which a country’s citizens are able to participate in selecting their government, freedom of expression, freedom of association, and a free media”. |
| **Rule of law** | Reflects the perceptions of the extent to which “agents have confidence in and abide by the rules of society”, and in particular the “quality of contract, property rights, the police and the courts, and the likelihood of crime and violence”. |
| **Control and corruption** | Reflects the perceptions of the “extent to which public power is exercised for private gain, including both petty and grand forms of corruption, as well as capture of the state by elites and private interests”. |
| **Political stability and the absence of violence and terrorism** | Measures perceptions of the likelihood of political instability and/or politically motivated violence. |
| **Government effectiveness** | Relates to the perceptions of the “quality of public services, the quality of civil service and the degree of its independence from political pressures, the quality of policy formulation and implementation, and the credibility of the government’s commitment to these policies”. |
| **Regulatory quality** | Relates to the perceptions of the ability of the government to formulate and implement sound policies and regulations that permit and promote public sector development. |
