# Supplementary material for: Shared governance increases marine protected area effectiveness
Source: PLoS One. 2025 Jan 8;20(1):e0315896. doi: 10.1371/journal.pone.0315896 (PMC11709245; doi:10.1371/journal.pone.0315896)
Supplement: S2 File — (DOCX) [file pone.0315896.s002.docx]

**S2 File. Posterior predictive distributions**


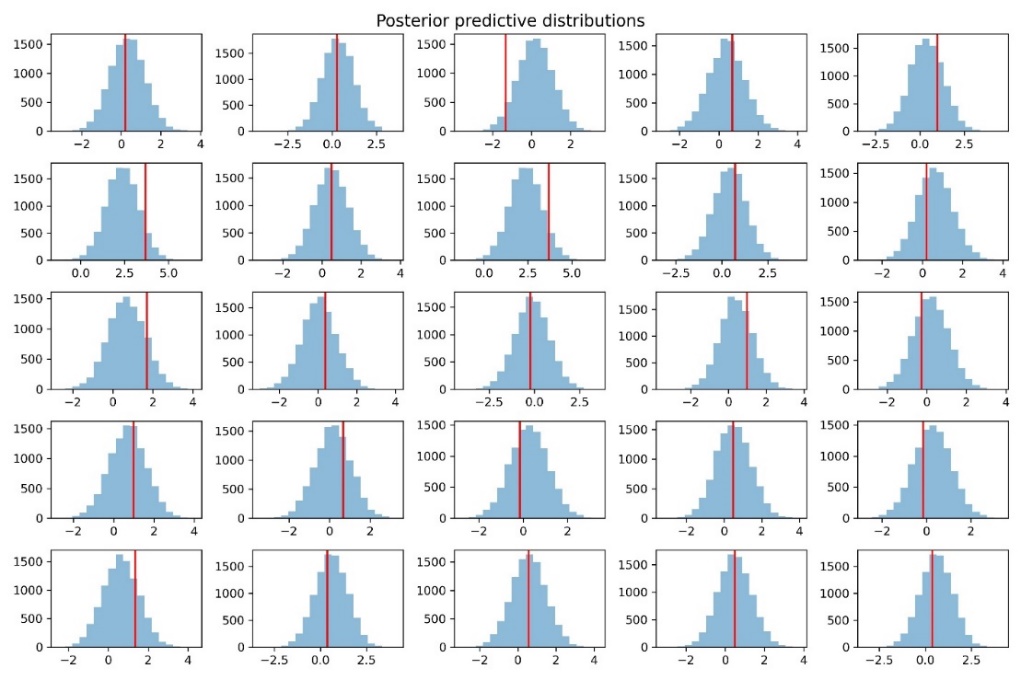


**Model 1 Posterior Predictive Distribution.** Posterior predictive distributions are shown for Model 1. Distributions that consistently overlap observed values are taken as evidence that Model 1 is consistent with the observed data and demonstrates that there is no evidence of poor model fit.


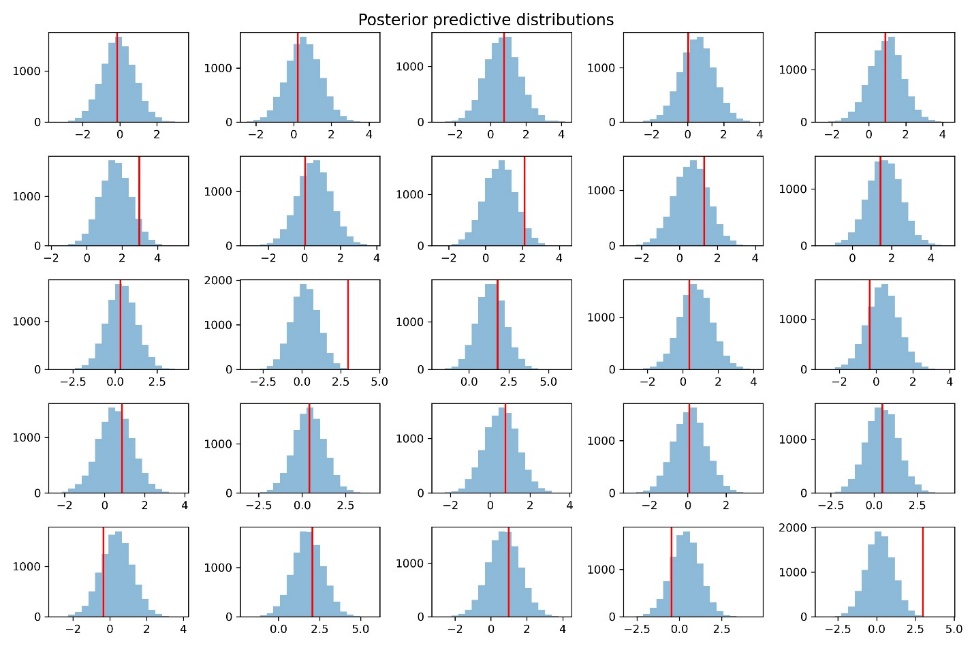


**Model 2 Posterior Predictive Distribution.** Posterior predictive distributions are shown for Model 2. Distributions that consistently overlap observed values are taken as evidence that the full Model 2 is consistent with the observed data and demonstrates that there is no evidence of poor model fit.
