# Supplementary material for: Shared governance increases marine protected area effectiveness
Source: PLoS One. 2025 Jan 8;20(1):e0315896. doi: 10.1371/journal.pone.0315896 (PMC11709245; doi:10.1371/journal.pone.0315896)
Supplement: S3 File — (DOCX) [file pone.0315896.s003.docx]

**S3 File.** **Posterior predictive distribution of the mean and Geweke Z scores**


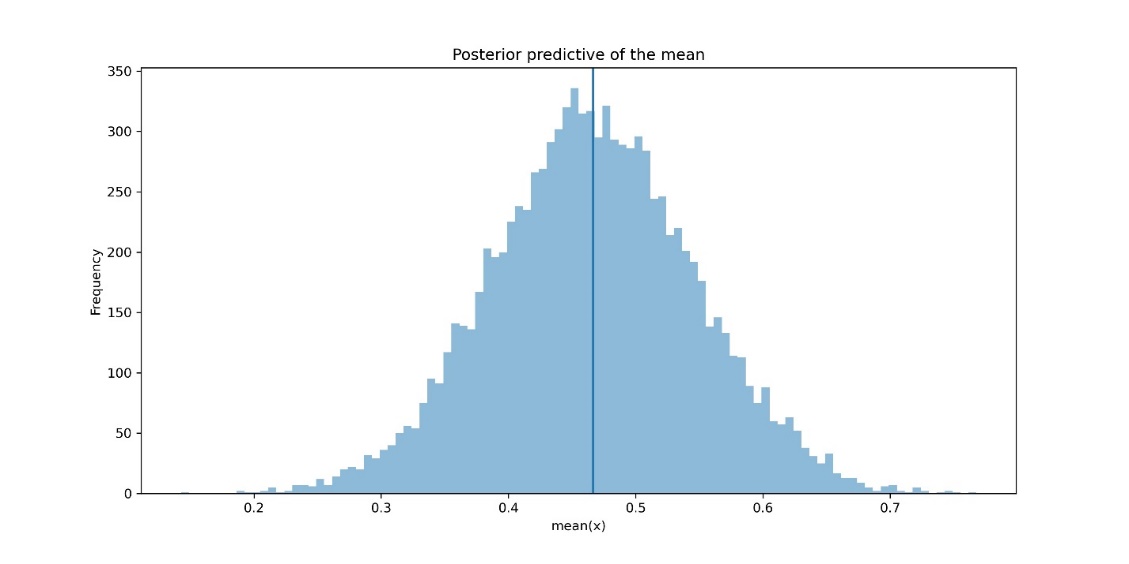


**S4 Fig.** **Model 1 posterior predictive distribution of the mean.** The posterior predictive distribution of the mean (blue histogram) shows no evidence of poor model fit. Geweke Z scores from observed fits of the model and data was 0.47 (shown in vertical blue line).


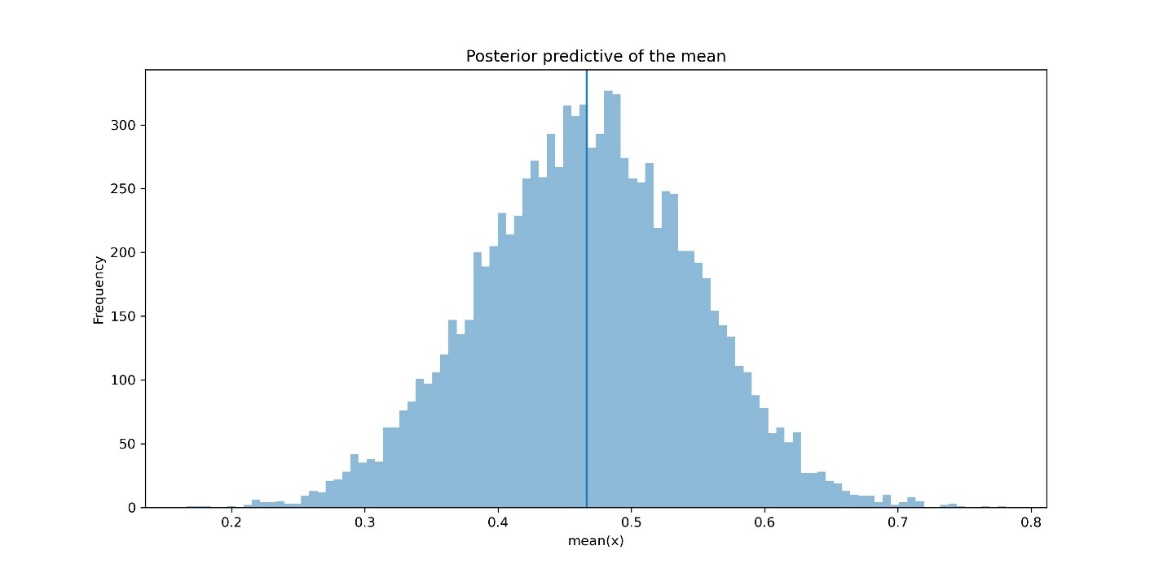


**S5 Fig. Model 2 posterior predictive distribution of the mean.** The posterior predictive distribution of the mean (blue histogram) shows no evidence of poor model fit. Geweke Z scores from observed fits of the model and data was 0.47 (shown in vertical blue line).
