# Supplementary material for: Shared governance increases marine protected area effectiveness
Source: PLoS One. 2025 Jan 8;20(1):e0315896. doi: 10.1371/journal.pone.0315896 (PMC11709245; doi:10.1371/journal.pone.0315896)
Supplement: S1 Fig — (DOCX) [file pone.0315896.s004.docx]

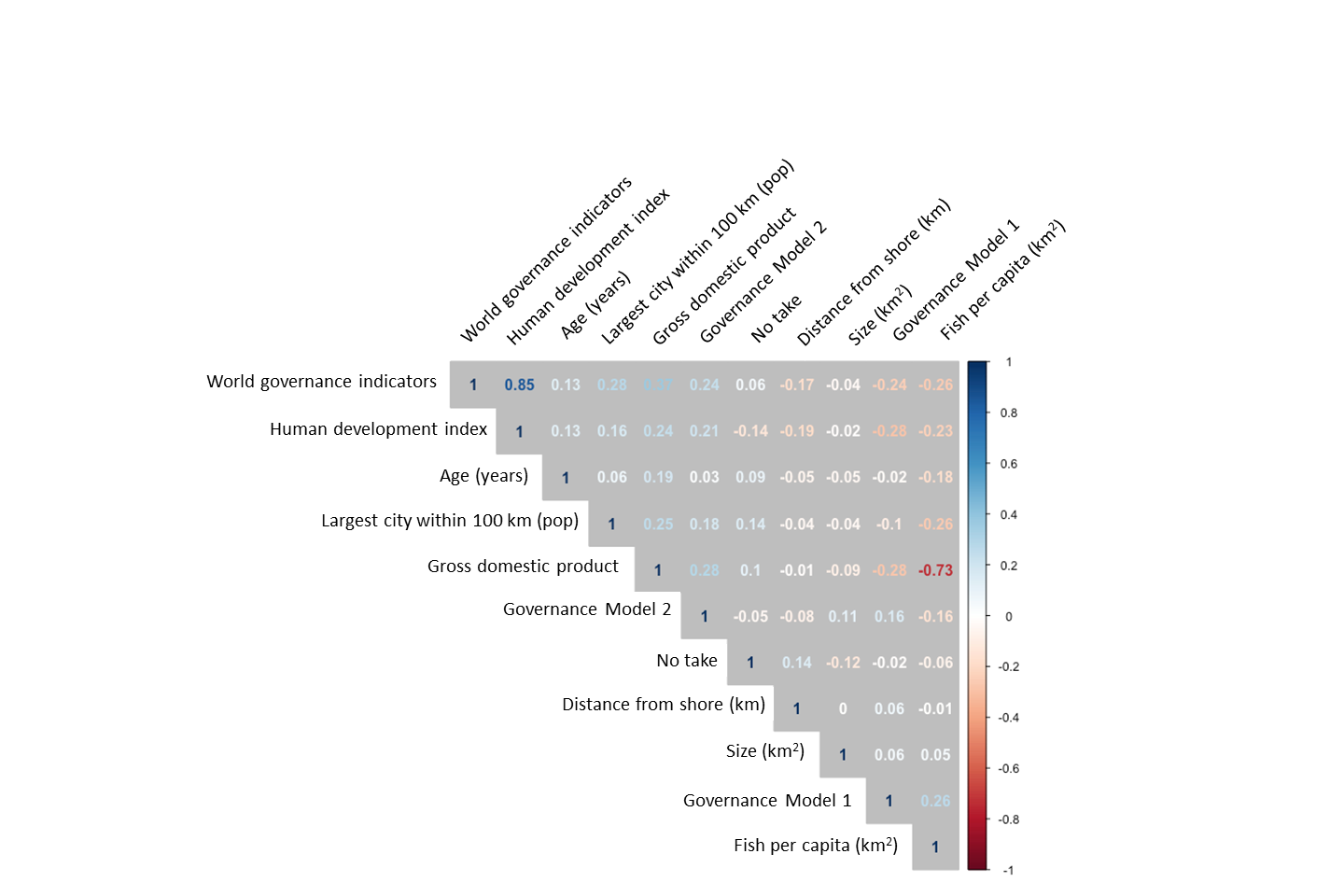


**S1 Fig.** **Correlation Matrix – Raw Data.** Correlation matrix for all covariates in Model 1 and Model 2, using the raw data. The number in each cell represents the results of a Pearson correlation test, ranging from 1 (positively correlated) to -1 (negatively correlated). Governance Model 1 includes the data classified as state or shared and Governance Model 2 includes the data classified as national, sub-national, collaborative, or joint.
