# Supplementary material for: Shared governance increases marine protected area effectiveness
Source: PLoS One. 2025 Jan 8;20(1):e0315896. doi: 10.1371/journal.pone.0315896 (PMC11709245; doi:10.1371/journal.pone.0315896)
Supplement: S2 Fig — (DOCX) [file pone.0315896.s005.docx]

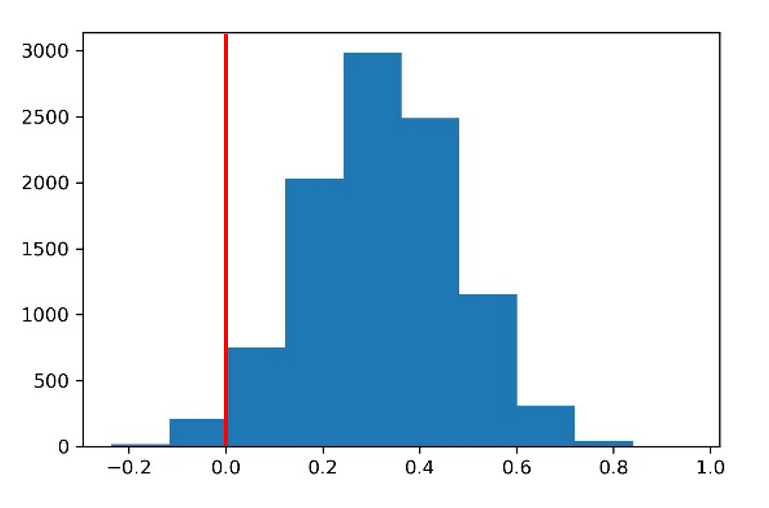


**S2 Fig.** **Shared Governance Posterior Density.** Histogram representing the proportion of the posterior density for shared governance that is above zero (red vertical line), given state governance is the baseline category. There was a 98% chance that shared governance would provide greater positive benefits than state governance.
