# Supplementary material for: Shared governance increases marine protected area effectiveness
Source: PLoS One. 2025 Jan 8;20(1):e0315896. doi: 10.1371/journal.pone.0315896 (PMC11709245; doi:10.1371/journal.pone.0315896)
Supplement: S3 Fig — (DOCX) [file pone.0315896.s006.docx]

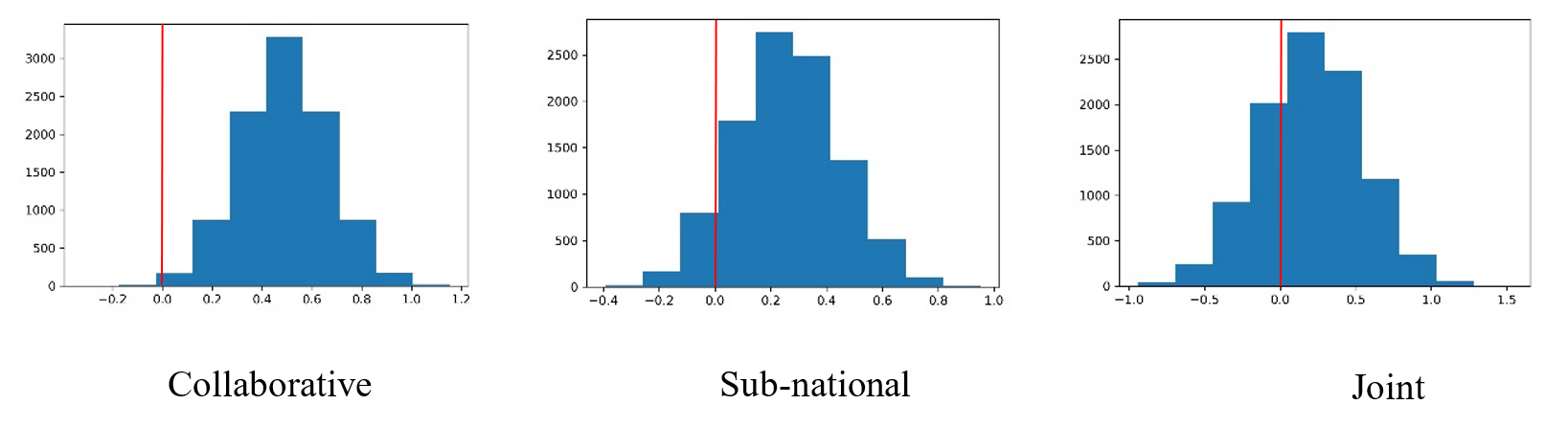


**S3 Fig.** **Collaborative, Sub-national and Joint Governance Posterior Densities.** Histograms showing the proportion of the posterior density of each governance type being above zero (red vertical line), given national governance as the baseline category. There was a 99%, 91%, and 73% chance that a collaboratively managed, sub-nationally managed, and a jointly managed MPA (respectively) would have greater biomass than a nationally managed MPA.
