# Supplementary material for: Shared governance increases marine protected area effectiveness
Source: PLoS One. 2025 Jan 8;20(1):e0315896. doi: 10.1371/journal.pone.0315896 (PMC11709245; doi:10.1371/journal.pone.0315896)
Supplement: S1 Table — (DOCX) [file pone.0315896.s007.docx]

**S1 Table.** **Covariate Mean Effect Sizes.** Mean effect sizes and 95% Bayesian credible intervals (C.I.) for each covariate in the model run without two of the correlated country-level covariates: World Governance Indicators and fish catch per capita per Exclusive economic zone (EEZ) area.

| Covariate |  | Effect size and 95% C.I. | |  |
| --- | --- | --- | --- | --- |
|  | **Model 2 without correlated covariates** | **Model 2 with correlated covariates** | **Model 1 without correlated covariates** | **Model 1 with correlated covariates** |
| MPA size (km^2^) | 0.061 + 0.31 | 0.06 + 0.30 | 0.056 + 0.3 | 0.05 + 0.31 |
| MPA age (years) | 0.24 + 0.24 | 0.21 + 0.24 | 0.21 + 0.24 | 0.24 + 0.24 |
| No take | 0.23 + 0.28 | 0.23 + 0.28 | 0.22 + 0.28 | 0.22 + 0.28 |
| Distance from shore (km) | 0.05 + 0.25 | 0.05 + 0.25 | 0.03 + 0.26 | 0.03 + 0.27 |
| Largest city within 100 km (pop) | -0.04 + 0.26 | -0.04 + 0.26 | -0.011 + 0.25 | -0.01 + 0.26 |
| Gross domestic product | 0.39 + 0.63 | 0.39 + 0.65 | 0.48 + 0.63 | 0.48 + 0.63 |
| Human development index | 0.046 + 0.32 | 0.04 + 0.32 | 0.026 + 0.33 | 0.04 + 0.32 |
| Shared governance | N/A | N/A | 0.32 + 0.31 | 0.32 + 0.30 |
| Collaborative governance | 0.49 + 0.35 | 0.49 + 0.35 | N/A | N/A |
| Sub-national governance | 0.26 + 0.37 | 0.25 + 0.37 |  |  |
| Joint governance | 0.21 + 0.65 | 0.21 + 0.66 |  |  |
